# Monthly Summary

**Published:** 1891-03

**Authors:** 


### Monthly Summary.

HÆMORRHAGE AFTER TOOTH EXTRACTION AND THE IMMEDIATE TREATMENT.—In order to intelligently treat a case, it is essential to ascertain if the blood that is being lost is or is not coagulable. To ascertain this, a little of the blood is caught in a cup; a firm clot will form in two or three minutes, if it is coagulable. For coagulable blood his treatment is with a styptic, or with a plug, with or without styptic. This cautery is his last resort and is only called for in case of non-coagulable blood. He considers nitric acid as the best styptic, but does not use it except other more convenient ones fail. He invented styptic colloid, which is composed of tannin in collodion, with addition of tincture of benzoin; and for ordinary cases he knows of none better. The colloid can be applied on cotton-wool and a firm plug of it inserted with much facility. He makes three kinds of plugs. *One* made of soft, well-teased wool saturated with the styptic colloid. A second plan is to use perchloride of iron, saturating cotton-wool with the solution and then drying the cotton down. He has also constructed what may be called the *wick styptic*, very useful in minor cases. It consists of a glass tube having a narrow point, through which is drawn a piece of cotton wick saturated with perchloride. A portion of the wick projecting from the narrow end of the tube can easily be inserted and firmly held in the cavity; when it becomes saturated with blood it can be cut off, a new bit pulled out and a fresh application made. He considers the best plug: one consisting of gutta-percha saturated with styptic, tannin or perchloride of iron. One of these disc, picked up by the forceps and held for a short time in hot water, softens, and becomes so malleable that it can be inserted into any cavity. The bleeding socket is quickly dried with absorbent cotton, and is then filled with the plastic styptic, pressed firmly down to the bottom and held there until it becomes firm. There is no fear of putting in the plastic styptic too hot, since heat favors coagulation. Presuming that the blood flowing from the injured part shows no tendency to clot, and is discharged in a steady stream which is *not* arrested by pressure or the ordinary applications, it is necessary at once to produce secondary or albuminoid coagulation. It must be remembered that no blood is constituted that cannot be made to coagulate. The electric cautery, if at hand, is the most convenient. The next best is a small iron cautery, an iron bulb terminating in a rounded point and fixed at an oblique angle in a strong handle. This bulb and point can be made red-hot in the flame of a spirit lamp or gas jet. It may be allowed to cool down until redness has disappeared, and can be inserted deep down into the bleeding alveolus; it should never be used at such a heat as to destroy structure. The coagulating power of heat over albumen, viz., 140 F., is all that is required, but this may be safely exceeded to 150 or 160 degrees. If all these means fail, God help the patient. He cautions against the use of stimulants, faintness being an advantage. The blood swallowed causes no harm—it is either digested or vomited. After treatment, mainly constitutional, he leaves to the physician. One form of plug used he does not mention, the plaster of paris. Last winter we had a case in which it had the desired effect. An inferior molar had been extracted, severe hæmorrhage followed; plaster of paris was mixed, quite thick, placed in the socket and allowed to extend over the proximal teeth; when hard it was trimmed smooth. The next day the patient removed the plug, hæmorrhage set in, the plug was cleaned, replaced, with a cessation of the flow. After two or three days the plug was left out, the case being cured.—*Dr. B. W. Richardson, in British Dental Journal.*

---

DENTAL PROSTHESIS.—"Prosthesis" is defined by Webster as follows: "In surgery, the addition of an artificial part to supply a defect of the body, as a wooden leg, etc." If we make a similar use of the word in dentistry, then dental prosthesis must mean the restoring of a dental defect by an artificial part, which would include, of necessity, fillings, crowns, bridges, plates, obturators, etc. This would divide the practice of dentistry into three branches —dental medicine, dental surgery, and dental prosthesis— the first to include all treatment depending on medicine; the second, surgery; the third, as indicated in the foregoing.

Dental prosthesis has had its fall, and now is on the up-grade, having greatly advanced since the advent of crowns and bridges. The introduction of rubber was the cause of its fall. Since the adoption of crowns and bridges, students take more interest in this branch, as there is a demand for such operations in all the land. The making of crowns calls for a knowledge of the working of gold, and it is but a step from making crowns to gold plates; hence, the recent graduates will take more kindly to the old regime, regarding plate-work in vogue previous to the advent of rubber. There was a time, shortly after the coming of rubber, when the maker of artificial substitutes for the human teeth was looked upon as being but a mechanic, and the idea of separating the two branches was seriously advocated, but that is all changed since the era of crowns, the connecting link between mechanical and operative dentistry, as then existing. Just why mechanical dentistry, a misnomer, should ever have been looked down on by the operative dentistry, is hard to comprehend by one who is an all-round dentist.

Those who have practiced general dentistry know that it requires more ability to insert a satisfactory artificial set of teeth than to fill a tooth with gold.

Dental prosthesis requires that the operator must be a good mechanic, an artist, physiologist, anatomist and physiognomist: a mechanic and artist, in order that he may be able to select the proper teeth and arrange them to harmonize with the natural parts; a physiologist, in order to understand the action of the oral muscles; an anatomist, to know them; a physiognomist, to understand the expression of the human face. The number practicing dentistry who fulfill all these requirements are few. This is proven by the failures, evidenced by actual observation in our daily contact with those who are disfigured by artificial teeth, often correctly called "false teeth."

---

TEETH IN DIABETES.—The importance of early diagnosis in diabetes cannot be over-estimated, because the prognosis may be much influenced by the institution of early treatment. It is interesting, therefore, to call attention to the fact that the *Wiener medizinische Presse*, January 4 1891, contains a brief abstract of a publication by Magitot in *Bulletin medical*, in which he dwells upon the diagnostic significance of alveolar osteo-periostitis as a manifestation of diabetes. He has noticed, in this disease, a deviation of the tooth, which, later, becomes loosened. At the same time an alveolar catarrh exists. Ultimately the tooth falls out. In the further course of the disease, the alveolar border of the bone may be absorbed, and this in turn may be followed by gangrene of the gum.—*Medical and Surg. Reporter.*

